# Telemedicine in sleep-related breathing disorders and treatment with positive airway pressure devices. Learnings from SARS-CoV-2 pandemic times

**DOI:** 10.5935/1984-0063.20210035

**Published:** 2022

**Authors:** Carlos Maria Franceschini, Marcela Viviana Smurra

**Affiliations:** 1 Hospital Cosme Argerich Gobierno de la Ciudad de Buenos Aires, Sleep and Mechanical Ventilation Unit, Intensive Therapy Division - Caba - Ciudad Autónoma de Buenos Aires - Argentina.; 2 Hospital Enrique Tornú. Gobierno de la Ciudad de Buenos Aires, Sleep and Respiratory Failure Laboratory, Pneumonology Service - Caba - Ciudad Autónoma de Buenos Aires - Argentina.

**Keywords:** Sleep Apnea, Obstructive, Continuous Positive Airway Pressure, Non-invasive Ventilation, Telemonitoring, Polysomnography, Telemedicine

## Abstract

The healthcare system currently faces new challenges, which are to be addressed by finding efficient alternatives. Such factors as the growth of world population, the increase in longevity, and the fact that some diseases which used to be deadly diseases have turned into chronic pathologies, cause the number of people in need for continuous medical care to rise. This results in a healthcare system crisis, which searches for solutions as telemedicine to address the needs of patients and control excessive medical spending. Telemedicine means remote medical assistance delivered by means of technological resources, which streamline the provision of medical care, thus increasing patient’s access to healthcare and saving time and costs. As regards respiratory diseases, telemedicine is a tool that may provide for proper prevention, diagnosis, therapeutic education, monitoring of observance, and therapeutic efficacy, as well as for the early detection of exacerbations. Patients suffering from sleep-related respiratory disorders in need for positive airway pressure devices may be benefited by telemedicine to enhance positive pressure adherence and follow-up to treat their pathologies, thus providing for the delivery of remote care and follow-up, reducing costs, and increasing the chances of receiving attention from specialists in patients who live a long distance from such medical facilities. However, it is a challenging task to find a balance in the doctor-patient virtual relationship.

## TELEMEDICINE

### History of telemedicine

The origin of telemedicine is indissolubly-related to telecommunications; i.e., sending information over long distances by means of electromagnetic signals. The arrival of the telegraph at the beginning of the 19^th^ century allowed for communication over long distances. The telegraph was used by telemedicine in military scenarios as a result of its transmission speed. The telegraph was used during the Civil War, in the United States, to ask for medical supplies, and to report deaths and injured soldiers on the battlefield. It seems likely that the telegraph could have also been used to ask for medical advice.

By the end of the 19^th^ century, Bell patented the telephone, but it was not until the early 20^th^ century that ordinary people could gain access to it on a massive scale. Back then, doctors and patients could talk directly on the phone. Medical providers could also speak on the phone with other physicians in order to ask for their expert opinion or to exchange information.

The first notion of telemedicine - as we know it today — was introduced in a Radio News Magazine edition from April 1924, which featured a futuristic illustration of a machine with a microphone and a television, which enabled patients to get in touch with their doctors. The device also included the use of temperature and heartbeat indicators. However, the first uses of telemedicine to broadcast videos, images, and complex medical data occurred in the late 1950s and in the early 1960s. In 1959, the University of Nebraska used interactive telemedicine to broadcast neurological exams. Such technology was originally developed to connect patients living in remote areas with physicians residing in urban areas.

Radiology was the first branch of healthcare to fully embrace telemedicine with the purpose of digitally broadcasting radiological images. Over the 1960s and 1970s, telemedicine was strongly boosted thanks to NASA research programs, since astronauts were not able to travel with a doctor by their side.

With the Internet booming, in the 1990s, there was an outburst of information. There was a true revolution in telemedicine that included patient education, broadcast of medical imaging, real-time audio and video medical consultations, and vital sign measurements.

### An overview of telemedicine

After having reviewed the report on mHealth, the 71^st^ World Health Assembly urges member states:

1. To assess their use of digital technologies for health, particularly in health information systems in the national and subnational levels, in order to identify areas of improvement, and to prioritize, when appropriate, the development, evaluation, implementation, extensions, and greater use of digital technologies, as a mean to promote fair, affordable, and universal access to health for everyone, as well as the special needs of groups that are vulnerable in the context of digital health;
2. To consider, when appropriate, how digital technologies could be integrated into existing health system infrastructures and regulations, to reinforce national and global health priorities by optimizing existing platforms and services, to promote people-centered health and disease prevention, and in order to reduce the burden on health systems;
3. To optimize the use of resources by means of developing health services alongside the application and use of digital technologies in health systems development and reforms;
4. To identify priority areas where normative guidance, technical assistance and advice on digital health would be beneficial, including, but not limited to: gaps in research, evidence-based standards, implementation and extension support, funding and business models, content, evaluation, cost-effectiveness and sustainability, data security, ethical and legal issues, the re-use and adaptation of existing digital health tools and other relevant ones as well;
5. To work towards and support the compatibility of digital technologies for health by, among other options, promoting the use of international and open standards as an affordable, effective and easily adaptable solution;
6. To spread, when appropriate, the best practices and successful examples of digital health architecture, programs, and services, in particular effective policy designs and practical implementations, among the international community, including through WHO, bilateral, regional, cross-regional and global networks, digital platforms and hubs;
7. To strengthen public health resilience and to promote opportunities, when appropriate, through the use of digital technologies, which includes improving access to, and monitoring, sharing and the use of, quality data, direct citizen, health care workers and government engagement, and to build the capacity for rapid response to disease incidents and public health emergencies, leveraging the potential of digital information and communication technology to enable multidirectional communications, feedback loops and data-driven “adaptive management”;
8. To build, especially through digital means, capacity for human resources in digital health, when appropriate, across both the health and the technology segments, and to communicate areas of specific need to the WHO in order to receive appropriate technical assistance;
9. To improve the digital skills of all citizens, in particular by working with the civil society to build public trust and support for digital health solutions, and to promote the enforcement of digital health technology in benefit of, and with access to, everyday health services;
10. To develop, when appropriate, the legislation and/or data protection policies around issues such as data access, sharing, consent, security, privacy, compatibility and inclusivity consistent with international human rights obligations and to communicate these on a voluntary basis to the WHO;
11. To develop, when appropriate, and in coordination with existing and emerging regional hubs and support mechanisms, effective partnerships with stakeholders from across all sectors in the use of digital health^1^.

### Current legal framework applicable to the telematic activity in Argentina

A study performed by the Argentina’s Ministry of Health, in 2015, about the distribution of physicians in Argentina described that, in spite of the fact that our country has a number of physicians per capita comparable with central countries, the distribution of physicians is highly unequal^2^.

Telehealth is a broader concept, which entails managing public health, medicine, health education and research. Such management is issued by communication and information technologies^3^.

As a result of the pandemic it was held that “telecare and/or teleconsultation” were to be interpreted as any remote consulting and/or assistance service by means of suitable technologies which guarantee service provision in a timely manner and subject to proper quality conditions, thus ensuring the prompt intervention in a health crisis context.

### Current applications of telemedicine

Reality and problems before and after the pandemic in Argentina’s telemedicine has shown a reduction in the number of people going to hospitals and emergency rooms in person, and in the costs, which result from such visits. The remote monitoring of patients reduces the number of unnecessary visits to hospitals and enhances communication among healthcare professionals, in addition to defining new roles of “experts in telemedicine”. Not only do these advantages impact on patients and on their caregivers, but they also impact on healthcare professionals and on management.

However, in spite of its potential advantages, it was slowly implemented before the COVID-19 pandemic, and it has found many “barriers” on its way. In order to understand this topic, it is necessary to analyze the standpoint of each “participant” that takes player” part in a telemedicine program.

There are only a few assignments that assess the opinions of healthcare professionals about the drawbacks they find in telemedicine, even though they all agree on the excessive workload as the main hindrance to overcome^4^. There are also some difficulties related to health funders issues to acknowledge the health services delivered, to invoicing the services rendered, and thus to the resulting loss of earnings, and the request of tests by these means.

The COVID-19 pandemic has introduced many necessary changes in the healthcare system, and telemedicine is one of the most important tools for communication, patient care, organization of virus testing scenarios, and patient isolation, expert consensus meetings, education and training, in relation to this serious situation that is affecting the entire world.

Healthcare professionals implemented such technology over the last few months as an essential tool for daily work, but it is highly probable that it may be here to stay in society as a big contribution to the current situation and face a future condition that may entail significant changes.

Patients had to learn to use this tool as their sole option to gain access to an outpatient consultation system during confinement. Patients started to adapt, and adhere, to the tool. The physician and his or her team prescribe a treatment to their patients, and they will have to suggest this key tool to improve remote follow-up and adherence^5-7^. The benefits of ongoing communication need to be explained in detail to the patients.

The studies conducted came to the conclusion that telemedicine is more profitable than standard care, and that patients are less satisfied with the doctor-patient relationship (DPR)^6,7^.

It is particularly important to provide patients with support when they start using TM, because TM is obviously different than traditional care in person. The first teleconsulting appointments will be the hardest to accept, until the appointment and monitoring paradigms change.

Informing patients of the benefits of this tool, where patients do not need to spend any waiting time for their medical appointments or pay for any travel allowances and sustain any loss of earnings in order to dismiss any doubts or to find solutions to any short-term treatment disadvantages.

There are TM implementation helpers. Evidence-based medicine states that patients with amyotrophic lateral sclerosis (ALS) and their caregivers and/or families show a positive attitude toward the use of telehealth^6,7^. This may be the result of the benefits obtained by patients (for example, a better qualification sense, a reduction in trip frequency and clinic load), and of good fulfillment with the use of TM (that is to say, easy-to-use devices, feeling comfortable with using technology, and providing caregivers with assistance). A positive attitude towards patients and caregivers acts as an implementation facilitator, because it increases system acceptance and positively impacts on the attitude of healthcare professionals.

There are some barriers to the implementation of TM. Some studies suggest that healthcare professionals have a more negative attitude towards telehealth, in spite of the fact that they have a positive outlook on communication through this tool. Most healthcare professionals reported barriers such as technical problems, lack of contact to make physical examinations, and problems with performing comprehensive medical evaluations. A negative attitude by healthcare professionals generates resistance to the system. This is something that has become a barrier when it comes to the application of implementing TM.

There are two very important telehealth implementation facilitators, which have positively influenced users’ attitude: training and continuous support. Customizing telehealth encourages patient involvement and it demands adjusting the monitoring frequency, scheduling visits with the treatment team, and providing care and information.

It is highly important that the healthcare team gets ready for telemedicine in order to enhance remote monitoring and the usage of monitoring data for research purposes. Standardized outcome measures (SOMs) must be set, and patients must take part in deciding which are the relevant measures^8,9^. In order to create healthcare teams in the field of telemedicine, it is necessary to reassign staff roles, increase the statistics and follow-up of patients’ safety, and to make the companies, which provide treatments with positive pressure devices to get involved.

### Telemedicine and sleep apnea

Obstructive sleep apneas (OSAs) are characterized by repetitive episodes of apnea-hypopnea caused by a collapse of the pharynx during sleep, causing desaturations and arousals. As these events become associated with a series of signs and symptoms, they amount to an obstructive sleep apnea-hypopnea syndrome (OSAHS)^10^. Nevertheless, a significant number of patients with OSA do not necessarily show any symptoms. Sleep apnea is a pathology with mortality and mainly cardiometabolic comorbidity. Mortality and life quality reduction are positively affected by the treatment with CPAP devices (positive airway pressure)^11,12^.

The use of this technological resource provides for:

- Telediagnosis;
- Teleconsultation;
- Remote monitoring;
- Medical meetings to get a second professional opinion (teleconference);
- Digital storage of data or medical records.

Sleep medicine uses the exchange of data with patients in order to better manage the disease. Data are conveyed over the Internet or over smartphones with specific applications considering the availability of feedback with the patient using the same resources. Telemedicine (TM) is useful for sleep apneas in all respects: from its detection to its treatment compliance by means of:

1. Remote polysomnography/polygraph tests subject to occasional distant controls by a technician;
2. Doctor-patient consultation;
3. Training on the use of devices for videoconference purposes;
4. Remote CPAP titration;
5. Video conference follow-up and compliance monitoring.

### Telediagnosis

The main goal is to obtain good quality records outside the sleep lab, to avoid long waiting lists at laboratories^13^.

Polygraph tests (RP): the use of home-based devices allowing for deferred data transmission is a possibility. Borsini et al. (2016)^14^ showed a 4 to 12% failure rate and the need for lab tests. This has shown that it is possible to deploy a decentralization strategy and to receive data promptly after recording. Always keeping in mind that type III studies, as RPs have a 64 to 100% sensitivity and a 41 to 100% specificity. Depending on the device, these features will enable us to set limitations to the method^15^.

Polysomnography (PSG) tests may be performed in patients’ homes or in any remote area, and they involve continuous or intermittent monitoring. Gagnadoux et al. (2002)^16^ performed a remote monitoring test in the care sector of peripheral hospitals and in patients’ homes by providing the nursing staff with training to correct any sensor placement mistakes. The fault percentage ranged from 11% to 23%, the latter pertaining to studies performed at home, and that therefore, they may be less monitored studies.

A work group in Belgium performed PSG tests with a device that had been specifically designed for telematic broadcasting purposes, added to the polysomnograph in order to transfer records to the sleep lab where technicians remotely monitor the tests. They communicate with patients via Skype to correct the position of any sensors, which may have shifted from their proper recording position. The SLEEP box device and the contact thru Skype made it possible to obtain 90% of good-quality signals^15^.

**Figure 1:**
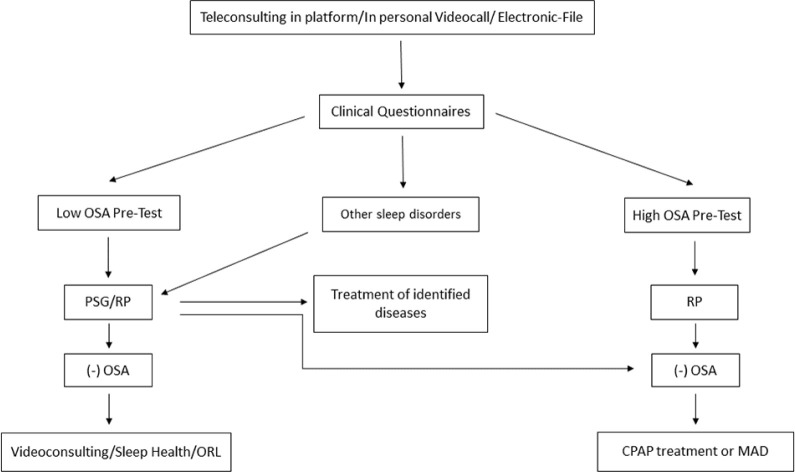
OSA Telediagnosis Flowchart. OSA: obstructive sleep apnea, PSG: polysomnography, RP: respiratory polygraphy, ORL: otorhinolaryngology, CPAP: continuous positive airway pressure, MAD: mandibular advancement device.

In a (maybe) near future, home-based polysomnography and polygraphy devices through telematic data transmission will be used, as well as smartphone-based breathing and environmental sensors, records of data collecting devices which gather information over several days, and contactless data transmission devices^16,17,18^ (Figure 1).

### Teleconsultation

Teleconsultation is a useful tool to explain the diagnosis and therapy follow-up of patients who have recently been evaluated over the Internet or by video conference call.

As it arises out of the work performed by Coma-del-Corral et al. (2013)^19^, the adherence to therapy had no significant differences in patients with a confirmed diagnosis who held a personal interview, as opposed to patients evaluated through teleconsulting, following the way of diagnostic information to CPAP titration.

### Treatment with CPAP devices

#### Beginning of the treatment with telemonitored CPAP

The first step in the treatment segment would consist of providing training on the device features, on the right use of the interface; and on how to use and manage the thermal humidifier, if it was prescribed to the patient. This telematic procedure could be supported by surveys addressed to patients in order to assess the patients’ level of understanding and the need to reevaluate actions.

An assignment describes remote CPAP titration by means of a telemetry unit on the CPAP device, which would prevent any probable calibration failures^19^. Remote calibration testing has not been set for the devices available to us^20^, except in delayed mode. See Table 1 steps in the use of telemedicine in sleep-related breathing disorders and treatment with positive airway pressure devices.

**Table 1:** Steps in the use of telemedicine in sleep-related breathing disorders and treatment with positive airway pressure devices.

Teleconsultation (First visit Follow-up)	The Epworth Sleepiness Scale, the STOP-BANG Questionnaire, and the Berlin Questionnaire are to be filled out by patients and uploaded to the platform. They may be sent before the teleconsultation session so that they are available during the first video visit.
	Neumonology TM Template: set on the teleconsultation platform.
**Telediagnosis**	Polygraphy (PR) tests conducted at patients’ homes and compressed data transmission by staff trained on data management for uploading purposes on a central server or platform to count on the studies for processing purposes. The file storage system is available even on different polygraphy devices. Patients will be able to receive a video that shows how to use the device to simplify its placement.
	Polysomnography (PSG) may be conducted as out-of-laboratory tests, and up to this date, there are no devices featured to transfer data on line. Two different suggestions can be made, as with PR tests: In the first place, remote recording demands that a technician place the sensors. Data collection may be performed on the following day. The data collected is zipped and sent to the platform. Another option to consider is to use an online centralized control with both computers being connected thru Team Viewer, which enables remote access to the record-taking and monitoring screen. A simple call can indicate anybody accompanying the patient how to correct any sensor positions^30^.
**CPAP Telecalibration**	A calibration study can be performed in any patient's home after having conducted the relevant training in person or by video visit and delivery of the devices to the patients to be used over 3 nights and according to each Laboratory's indications. The use of devices such as the Airsense (Resmed) or the Dreamstation (Philips -Respironics) provides access by means of specific software solutions, which allow for the assessment of cloud data or through a special segment for data transmission. Therefore, this allows access to fulfillment, pressures, and leak levels throughout the time of calibration.
	It would be ideal to access the data obtained from the cable-download monitoring devices or card-monitoring devices, which could be informed by the very patient by file transfer—via e-mail, for instance—to be thereafter downloaded to be interpreted in the Sleep Lab. In the latter case, were it not possible on these devices, the patient attendance in person would be required.
**Telemonitoring**	As it has been described in the previous section, 30, 60, and 90-day monitoring may be performed on the basis of the usual recommendations. At this point, it is worth stating that a personal visit may be scheduled, depending on the patients’ needs, and on the patients’ relationship with the relevant device and interfaces. Notifications may also be scheduled from the platform to be sent by e-mail or phone applications with consistent reminders on the use of the device in order to improve adherence, considering the possibility of separating patients with adaptation difficulties as opposed to those who can easily adapt.
	Telemonitored assessments may proactively contribute in the early detection of any adaptability difficulties.
**Tools for patients**	Medical device suppliers have designed specific applications for patients: My Air (Resmed) and Dream Mapper (Philips) would improve adaptability parameters if available, and provided that patients were able to use them.
	Provide for the access of patients to the platform so that they may upload or transfer their compliance data.
**Data**	It must be possible to store:
	1. Questionnaires: Epworth - Berlin - STOP-BANG
	2. PR and PSG with zip data in the platform or in a related server with access to the studies performed by means of each operator’s password
	3. Spreadsheets that show data obtained from the results of studies and calibration data with breakdown to be specified
	4. Access to the data spreadsheets to export them and create bases for research developments.

### Treatment follow-up

It is worth remembering the definition of adherence to therapy: 4 hours of nightly CPAP usage for 70% of the nights is considered adequate adherence to therapy. However, CPAP use above 6 hours/night contributed in reducing blood pressure and drowsiness^21^.

The early implementation of telematics strategies may be of essence for patient care and adjustment and such strategies currently allow for device programming.

**Figure 2:**
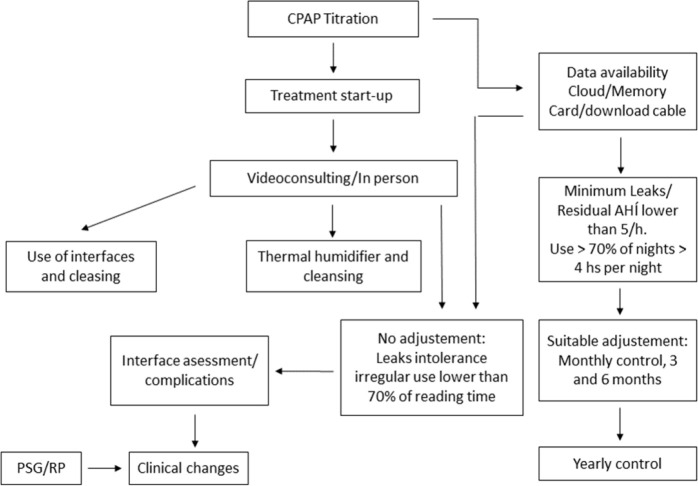
Commencement of OSA Treatment and Follow-Up Flowchart. CPAP: continuous positive airway pressure, PSG: polysomnography, RP: respiratory polygraphy, AHI: apnea hypopnea index.

The considerations to be evaluated are: adaptability and adherence, leaks, residual apnea index, and effective pressure. It has been found in studies assessing CPAP use for less than 4 hours per night or with leaks exceeding 0.5l/sec., telemonitoring outweighed traditional monitoring to restore efficient use time parameters^22^ (Figure 2).

It is also interesting that telemonitoring has further reduced the time for a first intervention by improving adherence in a short period of 3 weeks (5.7 as opposed to 4.2h/night). In that aspect, it is essential to have access to the data in order to be able to properly monitor compliance by patients with any difficulties to adapt over short periods of time and in the long term, and those patients who have easily adapted may be monitored with more time available^23^.

The repeated educational incentive by messages sent by email on days 1, 7, 14, 30, and 90 as opposed to a communication on the phone after 90 days derived in differences in the adherence to the CPAP therapy^24^.

Mobile phone apps provide patients with information on the daily use and patients may therefore access the information on the use of their device. The Spanish sleep group (*Grupo Español de Sueño*) has recently developed the *Appnea-Q* app, which generates questions (a total of 10), combining different aspects of treatment: CPAP use and effectiveness, common unfavorable effects, general topics such as: exercise, diet, and weight assessment. Patients receive tips, and encouraging messages^25,26^.

### Telemedicine and hypoventilation syndromes

#### Telemedicine for monitoring patients with non-invasive ventilation (NIV) support

Telemedicine in patients with alveolar hypoventilation subject to home-based non-invasive mechanical ventilation demands monitoring efficiency and adherence in non-dependent patients. In the case of dependent patients, teleassistance is additionally required for ventilation start-up, optimization of mechanical ventilation, and telesurveillance.

When we reviewed evidence-based telemedicine and its impact on respiratory diseases, we found randomized controlled clinical trials, as the trail performed by Pedone et al. (2013)^27^ which showed a drop in exacerbations and hospitalizations in older adult patients with COPD, using a multiparameter monitor by means of a cell phone with (SaO_2_, FC, Tºax) measurement.

Johnston et al. (2013)^28^ found a reduction in COPD severe exacerbations by using a software for the smart reporting of symptoms by telephone. Halpin et al. (2011)^29^ performed a randomized controlled trial of the effect of the automated interactive calls, combined with a health risk prognosis on the frequency and harshness of exacerbations in patients with clinically assessed COPD and using the EXACT PRO questionnaire.

Borel et a. (2015)^30^ showed that the software data from the NIV device could predict the onset of an exacerbation of COPD. They have also proved that the changes in the respiratory frequency and in the percentage of activated breathing transmitted via wireless connectivity were related to the onset of exacerbations in COPD.

In the context of the studies performed and pulmonary rehabilitation programs required to optimize NIV, we found that as per Marina et al. (2018)^31^, telemedicine represents a useful cost-effective tool, whose quality and effectiveness have been verified over time to perform quality telespirometry tests where considered necessary (distant healthcare centers without a specialist or in the patient’s home).

As regards the pulmonary tele-rehabilitation (PTR) of patients with COPD, Hansen et al. (2020)^32^ proved that the PTR did not outweigh the conventional pulmonary rehabilitation (CPR) in the 6-minute march test, and they did not find any differences among the groups, or as to the respiratory symptoms or life quality or physical activity or muscle function of lower limbs in patients with COPD and forced expiratory volume (FEV)_1_<50% in outpatients for day care facilities.

According to Paganoni et al. (2019)^33^, video televisits are possible visits, and they may be a useful tool to supplement the multidisciplinary care of patients with amyotrophic lateral sclerosis on non-invasive mechanical ventilation during sleep, video televisits provide considerable savings in adjusted costs for patients and institutions. Pinto et al. (2010)^34^ has managed to telemonitor the ventilators’ graphics in patients with amyotrophic lateral sclerosis, and to remotely modify the parameters, thus showing a reduction in the number of admissions to hospitals due to exacerbations and equal clinical progress.

Fernández-Granero et al. (2015)^35^ found that 75.8% of exacerbations in patients with COPD were early diagnosed by using an electronic stethoscope, which transfers auscultation sounds 5±1.9 days before the actual medical care, in average.

A study performed by Mansell et al. (2018)^9^ showed the optimization of non-invasive mechanical ventilation. The information concerning Tv (tidal volume), leak, FR respiratory rate, MV (minute ventilation), patient-triggered respirations, pressures reached, and patient’s adherence to therapy was downloaded via telemedicine and cards. Telemonitoring proved higher adherence and hours of ventilator use. The use of ventilator data downloading helped an early and objective assessment of the leaks and changes in ventilator parameters.

**Figure 3:**
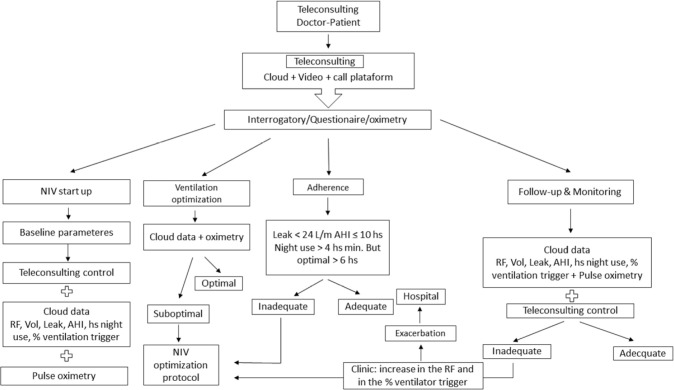
Telemedicine Flowchart for Non-Invasive Ventilation Support. NIV: noninvasive ventilation, AHI: apnea hypopnea index, RF: respiratory frequency, Vol: volume, hs: hours.

Through teleconsultation and telemonitoring, together with interrogation, questionnaires and oximetry, they allow to start non-invasive ventilation, optimize it, achieve adherence and carry out its follow-up monitoring (Figure 3).

The data obtained from the software of non-invasive mechanical ventilators are stored in smart cards or they may be read on the cloud through telemedicine. For instance, the apnea-hypopnea index recorded by the ventilator is safe and useful in order to optimize remote patient ventilation^36,37^.

The parameters set out below are the most widely used data downloaded via telemedicine or card to optimize home non-invasive mechanical ventilation:

- Tidal volume;
- Respiratory rate;
- Leaks due to inadequate interfaces or pressurization failure;
- Individual adherence with home NIV (residual AHI, average use hours per night, and leak);
- % of ventilator triggering by the patient.

### The objectives for patients in NIV

There are clinical improvement and reduction in daily PCO_2_, SatO_2_>90% 90% of time, minimal fluctuations in SatO_2_, monitoring in the NIV cloud, adherence, and fragmentation of ventilator use.

When these objectives are achieved, it is decided to continue with the same NIV parameters, otherwise unintentional leaks by the clinic in telemonitoring or data in the cloud must be ruled out.

**Figure 4:**
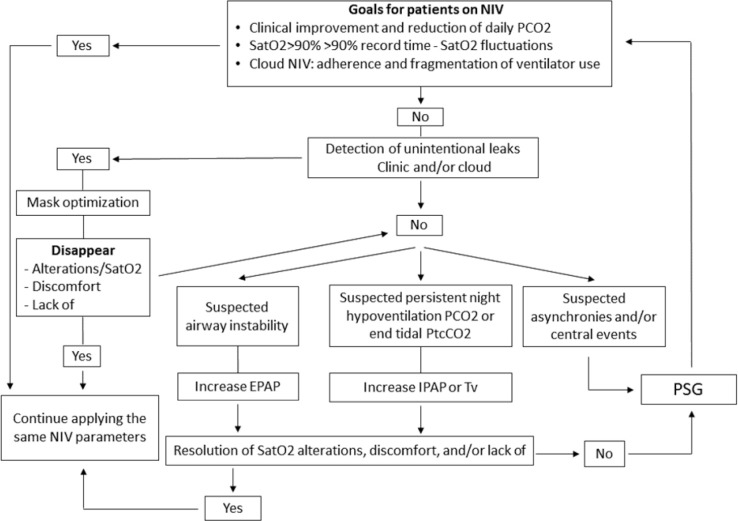
Optimization Flowchart. PCO2: partial pressure of carbon dioxide, SatO2: arterial oxygen saturation, NIV: noninvasive ventilation, PtcCO2: transcutaneous carbon dioxide pressure, EPAP: positive pressure in the airway at the end of expiration, IPAP: positive inspiratory pressure in the airway, PSG: polysomnography.

When there instability in the airway, an increase in EPAP is indicated, when suspected persistent night hypoventilation PCO_2_ or tidal PtcCO_2_, increase in IPAP or tidal volume and when there is suspicion of asynchronies and/or central events we indicate a polysomnography^38^ (Figure 4).

It is highly important that these diagnoses, adherence to therapy, follow-up, and telesurveillance parameters may be monitored and recorded by using telemedicine.

- BMI, Epworth daytime sleepiness scale, life quality questionnaire, and respiratory insufficiency questionnaire (SRI);
- Blood gases, end-tidal CO_2_ capnography, and transcutaneous pressure of CO_2_;
- Sleep study: AHI, oximetry (T90), IDO - I, II, III, and REM sleep phases;
- Interfaces: nasal, oronasal, total face, nasal pads, oral and mouthpiece;
- CPAP: pressure, autoCPAP: maximum and minimum pressure;
- NIV: modes S, S/T, PC, AVAPS. Parameters: IPAP, EPAP, RF, Tv, inspiration time (I/T), OXY monitoring, end tidal CO_2_, PtCO_2_;
- Leak, res AHI, Hs use/night, E Tv, inspiratory flow.

## DISCUSSION

According to the Pan American Health Organization (PAHO), it is estimated that one out of four individuals in America suffer from a non-communicable disease (NCD), including cardiovascular diseases, diabetes, cancer, and chronic respiratory diseases, which are the leading causes of death and disability in the world^39^.

Pursuant to recent surveys conducted by the World Health Organization-Pan American Health Organization (WHO-PAHO), from 31 to 53% of the NCD treatment services have been suspended - in whole or in part - in America, while primary healthcare services, which address 80% of health needs have been reduced by 20 to 30%.

In addition to that, 60% of diagnostic and medical practices have been canceled. On the basis of such data, the need arises to implement new innovative healthcare system models, which need to be explored in order to provide for uninterrupted medical care, including telemedicine, thus reducing stress in healthcare provision facilities.

The use of telemedicine technologies is a way to ensure the timely provision of medical services based on proper quality conditions. Within the framework of the current circumstances, healthcare professionals have no choice but to retrain on digital health in record time.

Telematic channels started to be used before the pandemic; however, the mobility crisis triggered by the pandemic clearly caused such channels to be used on a regular basis. The access mechanisms, the type and quality of communication, and the reconversion to person-to-person appointments, under specific circumstances must be ensured. Furthermore, from the legal standpoint, as any medical services, virtual medical appointments must be registered. Accordingly, follow-up mechanisms must be ensured and written indications and must be sent.

It is specifically with respect to sleep medicine that telemedicine developments enable access to diagnosis and treatment control, and the latter is a key option in performing the follow-up of home-ventilated patients. Based on the advantage that telemedicine had been developed prior to the health crisis, the pandemic provided for a faster and smoother implementation of telemedicine than in other medical areas.

Patients face many difficulties, such as long distance traveling, workload, loss of earnings, board and lodging costs, and family organization issues, among other factors, in order to be able to arrange medical appointments^40,41^.

In addition to those hindrances, there are other key advantages consisting in evading reduced mobility difficulties caused by disabling diseases. Confinement scenarios have currently extended such circumstances to the people at large, which have clearly benefited from a telematic access to non-urgent medical care.

Another important aspect that is worth mentioning, as far as medical practice is concerned, is the organization of the remuneration system by healthcare providers - in the particular case of private medical care providers -, which should have modified the payment for teleconsulting, drug and test prescriptions.

Data confidentiality and access to e-files must also be strictly monitored, and access codes must be generated for healthcare professionals at all times.
